# Cellular localization of protein arginine methyltransferase-5 correlates with grade of lung tumors

**DOI:** 10.1186/1746-1596-8-201

**Published:** 2013-12-10

**Authors:** Konstantin Shilo, Xin Wu, Smita Sharma, Meng Welliver, Wenrui Duan, Miguel Villalona-Calero, Junya Fukuoka, Said Sif, Robert Baiocchi, Charles L Hitchcock, Weiqiang Zhao, Gregory A Otterson

**Affiliations:** 1 Department of Pathology, The Wexner Medical Center at the Ohio State University, 410 W. 10th Avenue, Columbus, OH, 43210, USA; 2 Department of Medicine, The Wexner Medical Center at the Ohio State University, Columbus, OH, USA; 3 Department of Radiology, The Wexner Medical Center at the Ohio State University, Columbus, OH, USA; 4 Department of Pathology, Nagasaki University School of Medicine, Nagasaki, Japan; 5 Department of Molecular and Cellular Biochemistry, The Ohio State University, Columbus, OH, USA

**Keywords:** Protein arginine methyltransferase-5, Lung carcinoma, Neuroendocrine tumors

## Abstract

**Background:**

Protein arginine methyltransferase-5 (PRMT5) is a chromatin-modifying enzyme capable of methylating histone and non-histone proteins, and is involved in a wide range of cellular processes that range from transcriptional regulation to organelle biosynthesis. As such, its overexpression has been linked to tumor suppressor gene silencing, enhanced tumor cell growth and survival.

**Material and methods:**

Quantitative real-time polymerase chain reaction, Western immunoblot and immunohistochemistry were used to characterize PRMT5 expression in lung cancer cell lines and human tumors. Clinicopathological findings of tissue microarray based samples from 229 patients with non-small cell lung carcinomas (NSCLC) and 133 cases with pulmonary neuroendocrine tumors (NET) were analyzed with regard to nuclear and cytoplasmic PRMT5 expression.

**Results:**

There was statistically significant difference in PRMT5 messenger RNA expression between tumors and nonneoplastic lung tissues. Immunoblot experiments showed abundant expression of PRMT5 and its symmetric methylation mark H4R3 in lung carcinoma but not in non-neoplastic human pulmonary alveolar and bronchial epithelial cell lines. More than two thirds of lung tumors expressed PRMT5. High levels of cytoplasmic PRMT5 were detected in 20.5% of NSCLC and in 16.5% of NET; high levels of nuclear PRMT5 were detected in 38.0% of NSCLC and 24.0% of NET. Cytoplasmic PRMT5 was associated with high grade in both NSCLC and pulmonary NET while nuclear PRMT5 was more frequent in carcinoid tumors (p < 0.05).

**Conclusion:**

The observed findings support the role of PRMT5 in lung tumorigenesis and reflect its functional dichotomy in cellular compartments.

**Virtual slide:**

The virtual slides for this article can be found here: http://www.diagnosticpathology.diagnomx.eu/vs/1611895162102528

## Background

Lung cancer remains the greatest cause of cancer death, outnumbering the next three causes, colorectal, breast and prostate cancer combined. In 2011, it was estimated that more than 220,000 Americans would be diagnosed with lung cancer, and more than 150,000 people die from this disease [1]. Despite the development of targeted therapies directed against “driver” mutations in EGFR and ALK, nearly 50% of non-small cell lung carcinomas (NSCLC) currently lack identifiable mutations in these sites and therefore more targets beyond DNA mutation analysis including DNA methylation, mRNA and micro RNA expression analysis are a focus of investigations in lung cancer and other malignancies [2-6]. In particular DNA methylation of tumor suppressor genes has been found throughout entire spectrum of NSCLC and to be a nearly universal abnormality in squamous lung cancers [3,7,8].

DNA methylation of tumor suppressor genes was identified as having equivalent functional consequences as mutation of key tumor suppressor genes [9,10]. In contrast to mutations, DNA methylation is reversible, raising the possibility of “epigenetic” therapy by the use of hypomethylating agents to tumors with DNA hypermethylation. Two cytosine analogues that are incorporated into DNA during S-phase and block the maintenance DNA methylation machinery through the inhibition of DNA methyltransferase enzymes (DNMT) show benefit in myeloid tumors [11,12].

Similar to DNMT, protein arginine methyltransferase 5 (PRMT5) has been shown to be involved in silencing of tumor suppressor genes promoting neoplastic transformation. PRMT5, along with PRMT7 and PRMT9, forms a group of type II PRMT enzymes that catalyze symmetric arginine methylation of histones and non-histone proteins [13,14]. PRMT5 symmetrically methylates N-terminal of histones H3 (at Arginine position 8, S(Me2) H3R8), and H4 (at Arginine 3 S(Me2) H4R3), and leads to transcriptional silencing of regulatory and tumor suppressor genes [15]. Knockdown of PRMT5 leads to slower cellular growth, while over-expression of PRMT5 leads to cellular hyperproliferation. The overexpression of PRMT5 has been found in hematological and epithelial malignancies including lymphoma, prostate and lung cancer cell lines [14,16-18]. The cellular functions of PRMT5 are diverse and are, in part, related to nuclear or cytoplasmic localization (reviewed in [19]). In the nucleus, it is associated with several protein complexes including SWI/SNF chromatin remodelers. In the cytoplasm, PRMT5 forms a 20S protein arginine methyltransferase complex, termed the “methylosome,” consisting of spliceosomal snRNP Sm proteins, PRMT5, pICln, and WD repeat protein (MEP50/WD45) [20-22].

Since only limited data is available regarding the role PRMT5 in lung cancer, the goal of this study was to evaluate a large set of NSCLC and pulmonary neuroendocrine tumors (NET) for PRMT5 expression and the potential correlation of expression with clinicopathological variables.

## Material and methods

### Cell lines, resection specimens and tissue microarrays

NSCLC cell lines (NCI-H1299, NCI-A549, NCI-H520) and small cell lung carcinoma cell lines (NCI-H69 and NCI-H719) were obtained from American Type Culture Collection (Manassas, VA), Table 1. Non-malignant cell lines of human pulmonary alveolar epithelial cells (HPAEpiC) and human bronchial epithelial cells (HBEpiC) were obtained from ScienCell Research Laboratories (Carlsbad, CA). The cells were grown in routine media without cell cycle synchronization. The cell cultures were propagated for 7–14 days then collected and centrifuged and cell suspension pellets were either used for western immunoblotting or for immunohistochemistry (IHC) following paraffin embedding.

**Table 1:** Overview of employed materials

**Material**		**Features (n)**	**Source**
Cell line	NCI-H1299	Non-small cell lung carcinoma	American type culture collection (Manassas, VA)
	NCI-A549	Adenocarcinoma	
	NCI-H520	Squamous cell carcinoma	
	NCI-H69	Small cell lung carcinoma	
	NCI-H719	Small cell lung carcinoma	
	HPAEpiC	Human pulmonary alveolar epithelial cells	Sciencell research laboratories (Carlsbad, CA)
	HBEpiC	Human bronchial epithelial cells	
Frozen tissue		Adenocarcinoma (6)	Surgically resected tumors (Ohio State University Medical Center)
		Squamous cell carcinoma (2)	
Paraffin embedded tissue		Adenocarcinoma (3)	Surgically resected tumors (Ohio State University Medical Center)
		Squamous cell carcinoma (3)	
		Large cell neuroendocrine carcinoma	
		Small cell lung carcinoma (2)	
Tissue microarray		0.6 mm cores of non-small cell lung carcinoma (300)	Previously constructed [23,24]
		0.6 mm cores in duplicates of pulmonary neuroendocrine tumors (183)	

Six frozen tissue samples of adenocarcinoma (ADC) and 2 of squamous cell carcinoma (SQC) with matched nonneoplastic lung parenchyma (8 samples) were used for mRNA analysis. Three paraffin embedded samples of surgically resected lung ADC, 3 samples of SQC, 2 samples of small cell lung carcinoma (SCLC) and 1 large cell neuroendocrine carcinoma (LCNEC) were utilized for initial immunohistochemical analysis. The human tissues were collected in accordance with the institutional review board approved protocol.

Possible correlations between PRMT5 expression and clinicopathological variables were analyzed utilizing tissue microarrays (TMA) representing NSCLC and pulmonary NET previously constructed from the archival material of Armed Forces Institute of Pathology [23,24]. In short, the TMA comprised 0.6 mm cores obtained in duplicates from formalin-fixed paraffin-embedded tissue of 183 surgically resected pulmonary NET and 0.6 mm cores obtained from formalin-fixed paraffin-embedded tissue of 300 surgically resected NSCLC dating to the period from January 1980 to 2004. Eighty three tissue cores of non-neoplastic pulmonary parenchyma from the same cohorts served as normal control. After adjusting for core dropout, NSCLC TMA comprised clinical pathological annotations for 229 cases including 113 (49.3%) patients with ADC and 116 (50.7%) with SQC. Their median age was 65 years (range, from 36–86) including 24.4% females and 75.6% males. Staging information was available in 141 (61.6%) cases. Follow-up information was available in 189 (82.5%) cases with mean follow-up of 3.4 years (range from 0.1–14.2). NET TMA comprised annotations for 133 patients including 40 (30.1%) with typical carcinoid (TC) tumors, 23 (17.3%) with atypical carcinoid (AC) tumors, 19 (14.3%) with LCNEC, and 51 (38.4%) with SCLC. Their median age was 62 years (range, from 19–82) including 48.1% females and 51.9% males. Staging information was available in 100 (75.2%) cases. Follow-up information was available in 100 (75.2%) cases with mean follow-up of 4.3 years (range from 0.1–24.1). There were 83 samples of normal lung parenchyma included from the same patient population.

### Western immunoblot

Western immunoblot analysis was carried out according to standard protocol. Briefly, 30 μg of the total protein isolated from cells were used. Protein concentration was measured using a bicinchoninic acid protein assay kit (Pierce, Thermo Fisher Scientific, Rockford, IL). The primary antibody against PRMT5 (0.8 mg/ml) and Histone H4 (symmetric di methyl R3, 0.9 mg/ml) were obtained from Abcam (Cambridge, MA) and anti-β-actin from Santa Cruz Biotechnology (Santa Cruz, CA). The secondary antibody was horseradish peroxidase–conjugated goat anti-rabbit or mouse IgG. Proteins were detected using enhanced chemiluminescence and films (GE Healthcare).

### Real time polymerase chain reaction

Total RNA was isolated from frozen tissue using Trizol RNA isolation following the protocol supplied by the manufacturer (GIBCO BRL, Rockville, MD). RNA samples were treated with DNase (Ambion Inc, Austin, TX) to remove contaminating DNA and stored in -70°C freezer. Quantitation of mRNA expression was carried out using TaqMan real time polymerase chain reaction (PCR). Primers and probe used for the real time PCR analysis were obtained from Applied Biosystems (Foster City, CA). Reverse transcription was performed utilizing SuperScript II Reverse Transcriptase Kit (Applied Biosystems, Foster City, CA). Briefly, 500 ng of template total RNA was reverse transcribed in a 15 μl reaction. The PCR amplification was conducted in 25 μl reaction using the TaqMan Universal PCR Master Mixture (Applied Biosystems, Foster City, CA) according to the protocol supplied by the manufacturer. Real-time PCR was carried out in a 96-well plate using an Applied Biosystems 7900HT Sequence Detection System at 95°C for 10 minutes, followed by 40 cycles of 95°C for 15 seconds and 60°C for 1 minute. Each sample was evaluated in triplicate and each reaction was repeated at least once to ensure reproducibility. The PCR cycle number at threshold (CT) was used for the comparison. The relative quantitative method was used for the quantitative analysis. Calibrator was the averaged ΔCt from the non-tumor tissues. Endogenous control was ribosome RNA 18S gene (Applied Biosystems, Foster City, CA).

### Immunohistochemical analysis

IHC staining was performed in standard fashion on paraffin-embedded tissue. Paraffin embedded cell line pellet, resection and TMA block were cut at 4-microns and sections were placed on positively charged slides. Slides with sections were then placed in a 60°C oven for 1 hour, cooled, deparaffinized and rehydrated through xylenes and graded ethanol solutions to water. All slides were quenched for 5 minutes in a 3% hydrogen peroxide solution in water to block endogenous peroxidase. Slides then underwent heat-induced epitope retrieval employing Target Retrieval Solution (S1699, Dako, Carpinteria, CA) for 25 minutes at 96°C in a vegetable steamer (Black & Decker) and cooled for 15 minutes. Slides were then placed on a Dako Autostainer Immunostaining System. All incubations on the Autostainer were at room temperature. The primary rabbit polyclonal anti PRMT5 antibody (Abcam, Cambridge, MA) at 1:70 dilutions for tissues and at 1:150 for cell lines was incubated for 60 minutes. The two components of Mach 4 Universal Alkaline Phosphatase Polymer Kit (M4U536L, Biocare Medicals, Concord, CA) were applied sequentially for 15 minutes each. Staining was visualized with the Vulcan Fast Red chromogen (FR8055; 15 minutes development, Biocare Medicals, Concord, CA). Slides were then counterstained in Richard Allen hematoxylin (Thermo Scientific, Middletown, VA), dehydrated through graded ethanol solutions, cleared in xylene and coverslipped.

Based on the expression patterns identified in the resection specimens, the tumor cell staining in TMA was evaluated in comparison to normal alveolar parenchyma. Since cytoplasmic expression was evenly distributed within a tumor but varied in intensity, the cytoplasmic expression was assessed as negative (lack of staining), low (weak staining) and high (strong staining). Since number of PRMT5 expressing nuclei varied within a tumor, the nuclear expression was assessed as negative (lack of staining), low (staining in less than 50% of nuclei) and high (staining in 50-100% of nuclei).

### Statistical analysis

The mRNA expression levels were summarized graphically by plotting the mean and standard deviation of PRMT5 based on multiple amplifications. A *t* test was performed to compare the expression values in tumor samples with the values obtained from matched non-tumor samples. The associations between clinicopathologic variables and PRMT5 protein expression were examined with Pearson Chi-Square test. Survival probabilities were calculated using the Kaplan-Meier method and compared with the log-rank test. Prognostic significance of PRMT5 expression was evaluated with Cox regression model. Results with a p-value less than 0.05 were considered statistically significant. Data analysis was performed utilizing statistical package SYSTAT 13.0 (Systat Software Inc., Chicago, IL).

## Results

### PRMT5 is differentially expressed in malignant and normal lung tissue

**Figure 1:**
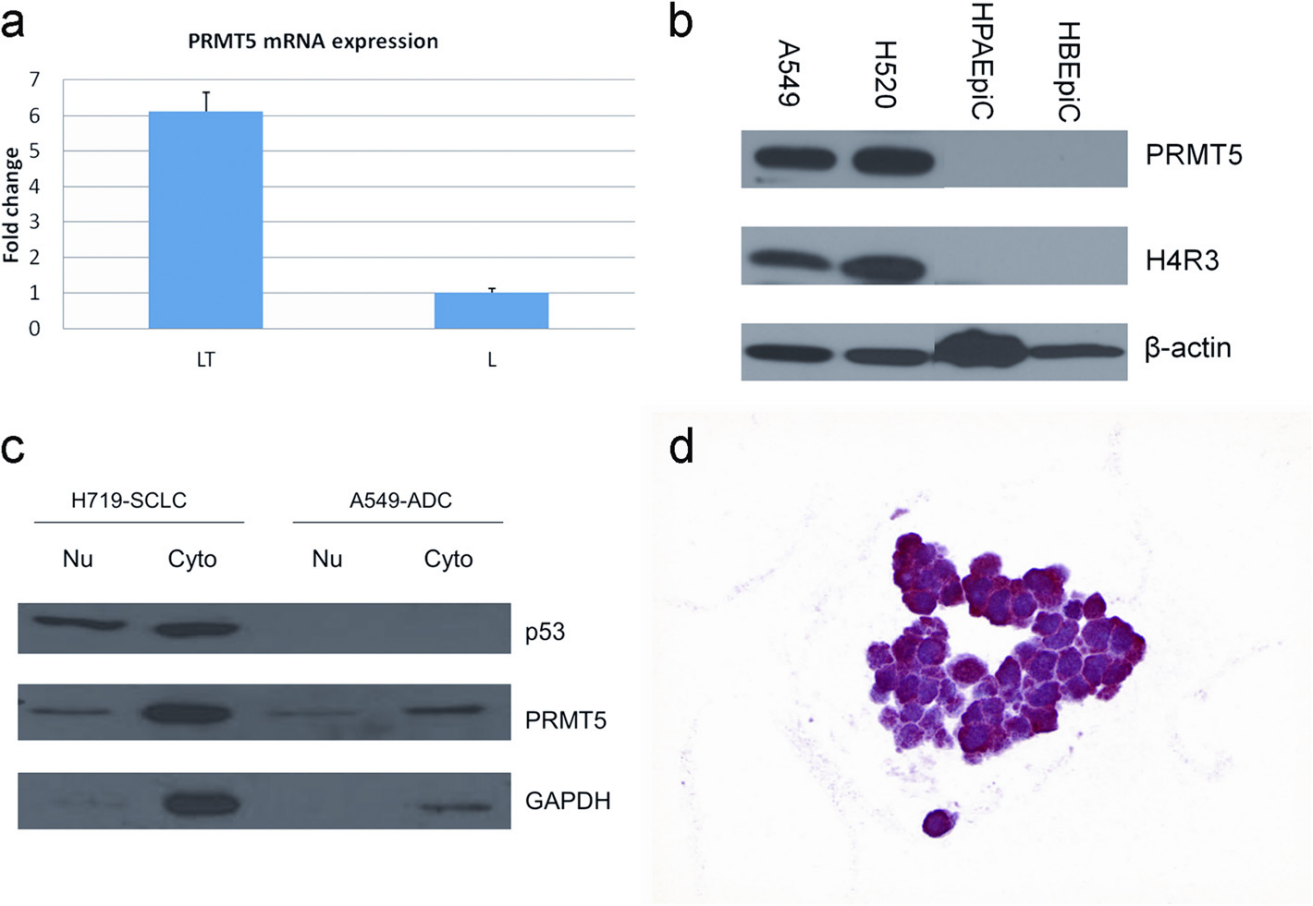
**PRMT5 overexpression in lung cancer is evident at mRNA and protein levels. (a)** There is a 6.13-fold increase in PRMT5 mRNA levels in lung tumors (LT), 8 cases, over matched nonneoplastic pulmonary parenchyma (L), as evident by TaqMan real time polymerase chain reaction (PCR). **(b)** PRMT5 and its symmetric methylation mark H4R3 are detected in lung carcinoma cell lines (NCI-A549, NCI-H520) but not in the nonneoplastic human pulmonary alveolar (HPAEpiC) and bronchial epithelial cell lines (HBEpiC); cellular localization experiments highlight nuclear and cytoplasmic fractions of PRMT5; Western immunoblot **(b, c)**; immunohistochemistry **(d)**, NCI-H69, original magnification ×600.

**Figure 2:**
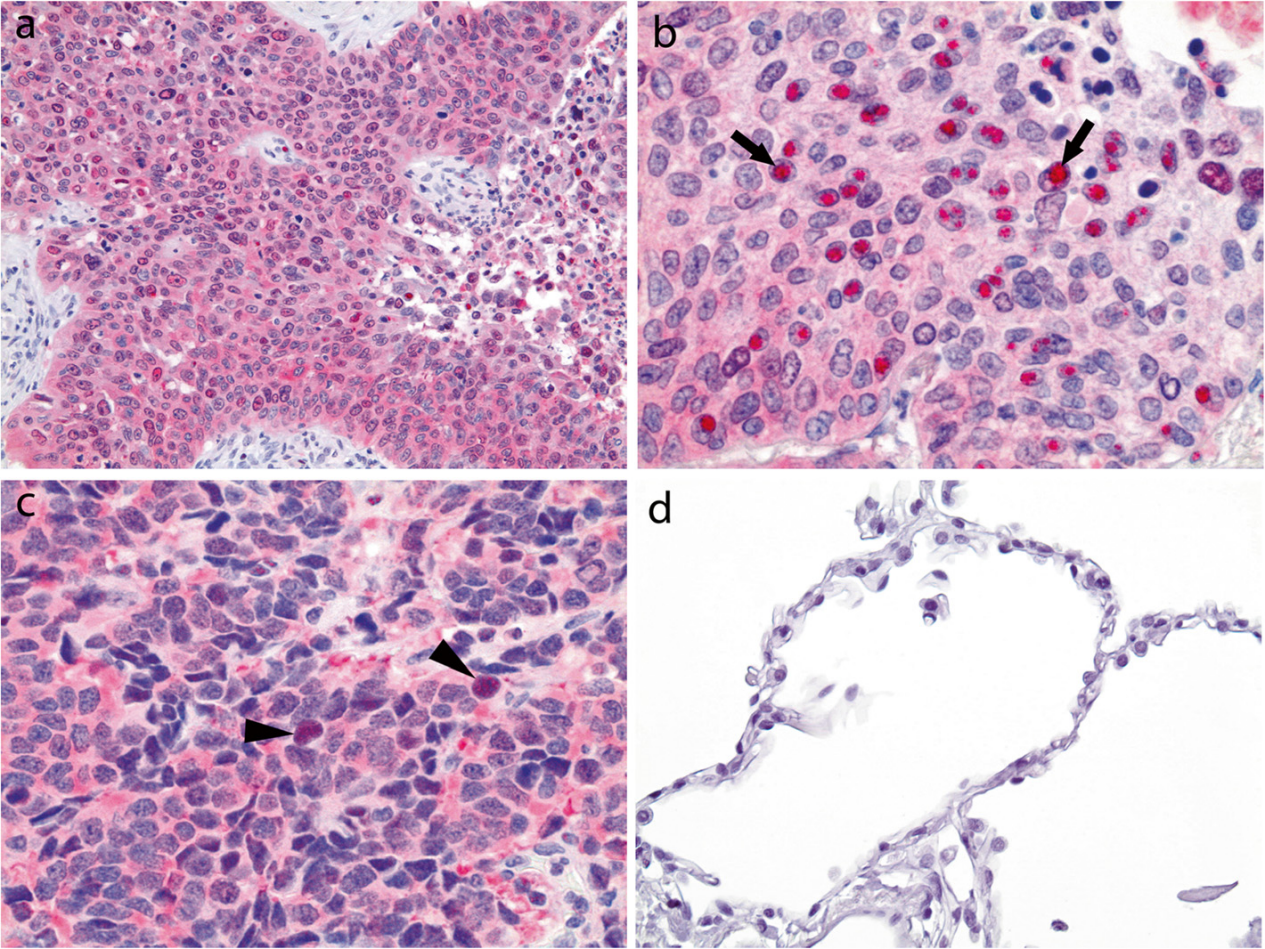
**Patterns of PRMT5 expression in surgically resected lung tumors.** Cytoplasmic PRMT5 is seen diffusely throughout a tumor **(a-c)** while nuclear expression varies **(b-c)**; squamous cell carcinoma **(a-b)**; small cell lung carcinoma **(c)**; immunohistochemistry, original magnification ×200, ×600, and ×600, respectively. A distinct nuclear accumulation of PRMT5 forming varying in size intranuclear globules is seen in squamous cell carcinoma, arrows **(b)**; in contrast, nuclear accumulation of PRMT5 in small cell lung carcinoma is finely granular, arrowheads **(c)**. No PRMT5 expression is seen in alveolar parenchyma away from tumors **(d)**, immunohistochemistry, original magnification ×400.

Mean PRMT5 mRNA evaluated with Taqman real time PCR was 6.13 fold higher in NSCLC samples than that in matched non-neoplastic pulmonary parenchyma, p = 0.030 (Figure 1a). Western immunoblot showed abundant expression of PRMT5 and its symmetric methylation mark S(Me2)H4R3 in lung ADC (NCI-A549) and SQC (NCI-H520) cell lines, but not in human pulmonary alveolar and bronchial epithelial cells (Figure 1b). Following protein fractionation, distinct bands of PRMT5 were seen in both cytoplasm and nucleus (Figure 1c). Cytoplasmic and nuclear expression of PRMT5 was identified in 5 of 5 lung carcinoma cell lines, including NSCLC (NCI-H1299), ADC (NCI-A549), SQC (NCI-H520), and SCLC (NCI-H69 and NCI-H719, Figure 1d). PRMT5 expression was also observed in 3 of 3 surgically resected ADC, 3 of 3 SQC, 2 of 2 SCLC and 1 LCNEC. All 9 carcinomas had cytoplasmic and nuclear PRMT5 expression. Most of the cells within resected tumors showed diffuse cytoplasmic PRMT5 while the number of PRMT5 expressing nuclei varied within a tumor (Figure 2a-c). In addition to diffuse cytoplasmic staining, NSCLC had a distinct nuclear accumulation of PRMT5 forming intranuclear globules (Figure 2b). In contrast, nuclear accumulation of PRMT5 in SCLC was finely granular (Figure 2c). Low level of nuclear PRMT5 was also seen in the alveolar parenchyma adjacent to tumors but not in the parenchyma (Figure 2d) away from tumors (8 of 8 cases).

### Cellular localization of PRMT5 correlates with tumor grade (differentiation)

**Figure 3:**
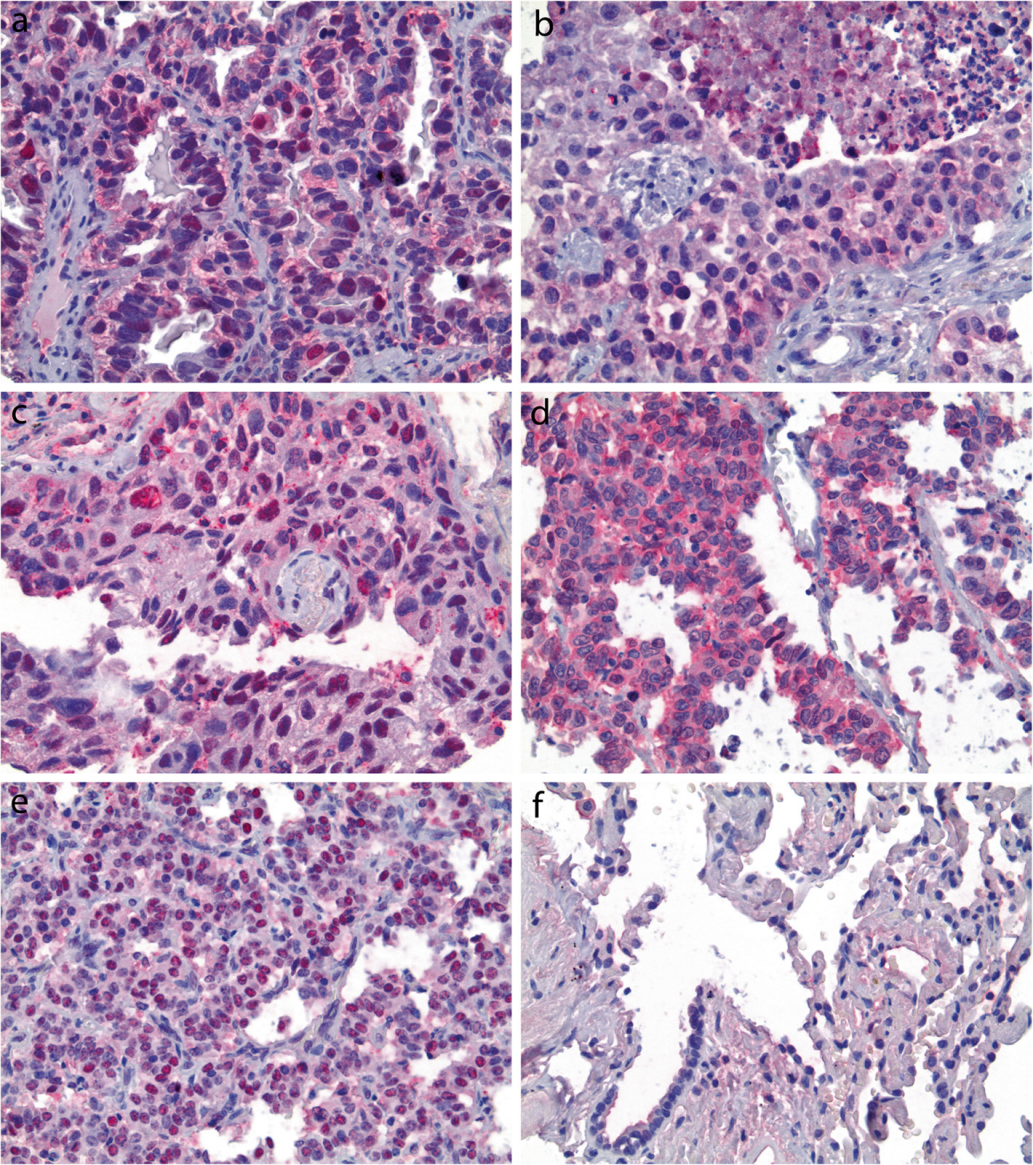
**Cytoplasmic (a-e) and nuclear (a, c and e) expression of PRMT5 is seen throughout the entire spectrum of lung tumors; representative examples of adenocarcinoma (a), squamous cell carcinoma (b-c), large cell neuroendocrine carcinoma (d) and typical carcinoid tumor (e); no PRMT5 is observed in normal alveolar parenchyma (f) Panels (b) and (d) illustrate low and high cytoplasmic expression, respectively; panels (a) and (e) illustrate low and high nuclear expression, respectively; immunohistochemistry; original magnification ×400.**

PRMT5 expression was present in the majority of NSCLC subjected to immunohistochemical analysis (Figure 3a-c). Cytoplasmic PRMT5 was detected in 66.4% (152 of 229) of cases, including in 20.5% (47 cases) at high levels. Nuclear PRMT5 was detected in 62.0% (142 of 229) cases including in 38.0% (87 cases) at high levels. Correlations between clinicopathological variables and PRMT5 expression in NSCLC are summarized in Table 2. High cytoplasmic PRMT5 was seen in 16.5% of poorly differentiated NSCLC versus 7.2% of well and moderately differentiated NSCLC, p = 0.01 (Table 2). High cytoplasmic PRMT5 was more common in SQC (12.2%) than ADC (8.3%), p = 0.04. High cytoplasmic PRMT5 correlated with grade of SQC but not ADC (data not shown). High nuclear PRMT5 was statistically more common in SQC (25.8%) than in ADC (12.2%), p < 0.001. No statistically significant correlation of cellular localization of PRMT5 with patients’ age, gender, tumor size, stage (Table 2) or outcome was identified in NSCLC (Tables 2 and 3).

**Table 2:** PRMT5 expression in NSCLC

		**Cytoplasmic high PRMT5, n (%)**	**P**	**Nuclear high PRMT5, n (%)**	**P**	**N**^**a**^
Age	≤60 years (n = 65)	11 (5.4)	0.36	22 (10.8)	0.55	204
	>60 years (n = 139)	30 (14.7)		55 (27.0)		
Gender	Female (n = 53)	9 (4.1)	0.79	19 (8.8)	0.25	217
	Male (n = 164)	35 (16.1)		62 (28.6)		
Size	≤3 cm (n = 74)	9 (6.3)	0.09	31 (21.8)	0.35	142
	>3 cm (n = 68)	18 (12.7)		22 (15.5)		
Stage	I (n = 81)	14 (9.9)	0.63	32 (22.7)	0.19	141
	II-IV (n = 60)	13 (9.2)		21 (14.9)		
Grade	WD, MD (n = 55)	7 (7.2)	0.01	20 (20.6)	0.98	97
	PD (n = 42)	16 (16.5)		16 (16.5)		
Histology	ADC (n = 113)	19 (8.3)	0.04	28 (12.2)	<0.001	229
	SQC (n = 116)	28 (12.2)		59 (25.8)		
Survival	Yes (n = 44)	9 (4.8)	0.37	14 (7.4)	0.54	189
	No (n = 145)	30 (15.9)		57 (30.2)		

*Abbreviations*: *Neg* negative, *N* number of cases, *WD* well differentiated, *MD* moderately differentiated, *PD* poorly differentiated, *ADC* adenocarcinoma, *SQC* squamous cell carcinoma.

^**a**^Reflects total number of cases with available annotations.

**Table 3:** Association of clinicopathological variables with overall survival in univariate and multivariate analyses (p-values; Cox-regression)

	**NSCLC**		**NET**		**Carcinoid tumors**		**HG NET**	
	**Uni**	**Multi**	**Uni**	**Multi**	**Uni**	**Multi**	**Uni**	**Multi**
PRMT5-Cyto	0.47	NI	0.99	NI	0.95	NI	0.59	NI
PRMT5-Nucl	0.62	NI	0.02	0.04	0.11	NI	0.98	NI
Age	0.84	NI	0.02	0.49	0.11	NI	0.88	NI
Gender	0.01	0.03	0.33	NI	0.74	NI	0.75	NI
Size	0.11	NI	0.71	NI	0.68	NI	0.62	NI
Grade	0.79	NI	0.000	0.000	0.001	0.001	0.81	NI
Stage	0.02	0.03	0.000	0.000	0.001	0.001	0.000	0.000

*Abbreviations*: *NET* neuroendocrine tumors, *HG-NET* high grade neuroendocrine tumors, *NI* not included, *NSCLC* non-small cell lung carcinoma, *Uni* univariate analysis, *Milti* multivariate analysis.

PRMT5 expression was also seen in the majority of pulmonary NET (Figure 3d-e). Cytoplasmic PRMT5 was detected in 88.0% (117 of 133 cases) of pulmonary NET including at high levels in 16.5% (22 cases). Nuclear PRMT5 was present in 60.9% (81 of 133 cases) including at high levels in 24.0% (32 cases). None of normal alveolar parenchyma tissue cores showed detectable levels of PRMT5 (0 of 83 cases, Figure 3f). Correlations between clinicopathological variables and PRMT5 expression in NET are summarized in Table 4. Cellular localization of PRMT5 correlated with tumor grade (differentiation), where high cytoplasmic PRMT5 was more frequent in high-grade NET (12.0%) than in carcinoid tumors (4.5%), p = 0.04, (Table 4). Conversely, high nuclear PRMT5 was statistically more common in carcinoid tumors (16.5%) than in high-grade NET (7.5%), p = 0.02, (Table 4). No statistically significant correlation of cellular localization of PRMT5 with patients’ age, gender, tumor size, or stage was identified in NET. Adjusted for tumor type, no statistically significant correlation of cellular localization of PRMT5 with outcome of pulmonary NET was seen (Tables 4 and 3).

**Table 4:** PRMT5 expression in NET

		**Cytoplasmic high PRMT5, n (%)**	**P**	**Nuclear high PRMT5, n (%)**	**P**	**N**^**a**^
Age	≤60 years (n = 59)	8 (6.3)	0.43	12 (9.4)	0.61	128
	>60 years (n = 69)	12 (9.4)		19 (14.8)		
Gender	Female (n = 62)	7 (5.4)	0.33	18 (14.0)	0.16	129
	Male (n = 67)	14 (10.9)		13 (10.1)		
Size	≤3 cm (n = 69)	9 (8.3)	0.71	19 (17.6)	0.76	108
	>3 cm (n = 39)	7 (6.5)		11 (10.2)		
Stage	I (n = 69)	12 (12.0)	0.48	17 (17.0)	0.42	100
	II-IV (n = 31)	7 (7.0)		10 (10.0)		
Histology	Carcinoid (n = 63)	6 (4.5)	0.04	22 (16.5)	0.02	133
	HG-NET (n = 70)	16 (12.0)		10 (7.5)		
Survival	Yes (n = 46)	8 (8.0)	0.923	16 (16.0)	0.02	100
	No (n = 54)	9 (9.0)		8 (8.0)		

*Abbreviations*: *Neg* negative, *N* number of cases, *HG-NET* high grade neuroendocrine tumors.

^**a**^Reflects total number of cases with available annotations.

## Discussion

Epigenetic regulation plays an important role in oncogenesis, and histone modification has been recognized as one strategy for modifying epigenetic controls [25,26]. It has been shown in cell culture and in animal models that PRMT5 is an important epigenetic modifier of histone and non-histone proteins in lymphomas, breast, colorectal and lung cancer, and its overexpression is associated with aggressive phenotype in these models [14,17,25,27,28]. In this study, we further corroborate the previous findings by showing a statistically significant difference in PRMT5 mRNA expression between tumors and matched nonneoplastic lung tissues in surgically resected specimens. We also show that the PRMT5 protein expression is significantly increased in lung cancer but not in nonneoplastic alveolar and bronchial epithelium cell lines. Furthermore, our investigation of a wide spectrum of lung tumors by immunohistochemistry confirms that consistent with quantitative real time PCR results, PRMT5 expression is present in a majority of tumors and its expression supports prior *in vitro* and *in vivo* studies suggesting importance of PRMT5 for proliferation of lung cancer cells.

Based on IHC analysis of more than 350 pulmonary tumors, our study also reveals that cytoplasmic PRMT5 is associated with higher grade in both NSCLC and pulmonary NET. Nuclear PRMT5 was more frequent in well-differentiated tumors (carcinoid tumors) than in poorly-differentiated tumors (SCLC and LCNEC). These findings further support the *in vitro* observations that cytoplasmic function of PRMT5 relates to silencing of multiple growth promoting and cell death inducing molecular targets [14,15]. Similar association was observed in prostate lesions [19]. Based on a subcellular localization assay, it was shown that in prostate cells, the functional activity of PRMT5 depends on its cellular location. In the cytoplasm, PRMT5 was essential for prostate cancer cell growth; in contrast, in the nucleus it inhibited cell growth. Accordingly, PRMT5 was preferentially expressed in nuclei of benign prostate tissues and in cytoplasm of premalignant and malignant lesions [18]. In light of accumulated findings, the differential expression of PRMT5 may speak to the distinct protein targets of this enzyme or to the shuttling of the enzyme in and out of the nucleus. In addition to histones, other intranuclear and cytoplasmic protein targets of PRMT5 have been suggested and demonstrated experimentally [13]. The predilection for cytoplasmic localization of PRMT5 in high-grade tumors may have implications for development of anticancer therapies inhibiting cytoplasmic targets including methylosome [29].

Our study identified that within NSCLC, both cytoplasmic and nuclear PRMT5 expression was more frequent in squamous cell carcinomas than in adenocarcinomas, which is in line with prior observations that DNA methylation is universal phenomenon in lung squamous cell carcinomas [7,8]. Cytoplasmic PRMT5 also correlated with grade of NSCLC but not outcome. Only tumor stage and female gender were statistically significant prognostic indicators in the study NSCLC cohort. Nuclear PRMT5 expression was associated with better outcome of all pulmonary NET; however, it was not independent of tumor type and the observed survival benefit was due to predilection of nuclear PRMT5 to carcinoid tumors exhibiting better prognosis.

It has been recognized that genetic instability is common in pulmonary parenchyma adjacent to tumors. Shared genetic changes have been documented in tumors, precursor lesions and non-neoplastic lung parenchyma adjacent to tumors [30]. Chromosome alterations can occur in non-neoplastic bronchial mucosa at a distance of 4 cm from the tumor boundary [31]. Therefore it is not surprising that in the current study, low levels of PRMT5 expression were noted in reactive tissues adjacent to tumors in resection specimens and not in alveolar parenchyma distant from tumors or normal controls in TMA. Similar to our study, low levels of PRMT5 have been reported at low frequency in normal ovarian tissues and benign ovarian tumors [32]. While PRMT5 overexpression and its cellular localization appear to be associated with more aggressive tumor phenotypes, alterations in PRMT5 expression alone may not necessarily lead to malignant transformation per se, but reflect changes of epigenetic control in oncogenesis. Evaluation of PRMT5 in a broader set of reactive and premalignant pulmonary lesions deserves further investigation.

In summary, our study shows that PRMT5 is significantly overexpressed in neoplastic lung tissues supporting its role in lung tumorigenesis. Cellular localization of PRMT5 correlates with lung tumor grade/differentiation, supporting functional dichotomy of PRMT5 in cellular compartments.

## Abbreviations

PRMT5: Protein arginine methyltransferase-5; HPAEpiC: Human pulmonary alveolar epithelial cells; HBEpiC: Human bronchial epithelial cells; TMA: Tissue microarray; NET: Neuroendocrine tumors; TC: Typical carcinoid; AC: Atypical carcinoid; LCNEC: Large cell neuroendocrine carcinoma; SCLC: Small cell lung carcinoma; ADC: Adenocarcinoma; SQC: Squamous cell carcinoma.

## Competing interests

The authors declare that they have no competing interests.

## Authors’ contributions

KS, SS, RB, GAO: conceived and designed the study; XW, SS, WD: performed the experiments; KS, XW, MW, WD: analyzed the data; MV-C, JF, CLH, WZ: contributed reagents/materials/analytic tools; KS, MW, GAO: wrote/edited the paper. All authors read and approved the final manuscript.
